# A Hybrid Model for Predicting the Prevalence of Schistosomiasis in Humans of Qianjiang City, China

**DOI:** 10.1371/journal.pone.0104875

**Published:** 2014-08-13

**Authors:** Lingling Zhou, Lijing Yu, Ying Wang, Zhouqin Lu, Lihong Tian, Li Tan, Yun Shi, Shaofa Nie, Li Liu

**Affiliations:** Department of Epidemiology and Biostatistics, School of Public Health, Tongji Medical College, Huazhong University of Science and Technology, Wuhan, China; The George Washington University Medical Center, United States of America

## Abstract

**Backgrounds/Objective:**

Schistosomiasis is still a major public health problem in China, despite the fact that the government has implemented a series of strategies to prevent and control the spread of the parasitic disease. Advanced warning and reliable forecasting can help policymakers to adjust and implement strategies more effectively, which will lead to the control and elimination of schistosomiasis. Our aim is to explore the application of a hybrid forecasting model to track the trends of the prevalence of schistosomiasis in humans, which provides a methodological basis for predicting and detecting schistosomiasis infection in endemic areas.

**Methods:**

A hybrid approach combining the autoregressive integrated moving average (ARIMA) model and the nonlinear autoregressive neural network (NARNN) model to forecast the prevalence of schistosomiasis in the future four years. Forecasting performance was compared between the hybrid ARIMA-NARNN model, and the single ARIMA or the single NARNN model.

**Results:**

The modelling mean square error (MSE), mean absolute error (MAE) and mean absolute percentage error (MAPE) of the ARIMA-NARNN model was 0.1869×10^−4^, 0.0029, 0.0419 with a corresponding testing error of 0.9375×10^−4^, 0.0081, 0.9064, respectively. These error values generated with the hybrid model were all lower than those obtained from the single ARIMA or NARNN model. The forecasting values were 0.75%, 0.80%, 0.76% and 0.77% in the future four years, which demonstrated a no-downward trend.

**Conclusion:**

The hybrid model has high quality prediction accuracy in the prevalence of schistosomiasis, which provides a methodological basis for future schistosomiasis monitoring and control strategies in the study area. It is worth attempting to utilize the hybrid detection scheme in other schistosomiasis-endemic areas including other infectious diseases.

## Introduction

Schistosomiasis is a neglected tropical parasitic disease caused by blood-flukes of the genus Schistosoma [1], [2], which is endemic in tropical and sub-tropical areas [3]. Schistosomiasis can lead to a series of acute or chronic symptoms including diarrhea, bowel ulceration, chronic pain, pulmonary hypertension, anaemia, undernutrition, and exercise intolerance in humans [4], [5]. It was estimated that more than 200 million individuals were infected, close to 800 million were at risk [6], and published disability-adjusted life-years exceeded 70 million [7]. According to the World Health Organization, 243,192,887 people required treatment for schistosomiasis in 2011 [8]. Hence, the prevalence of schistosome infection undermines social and economic development in the affected countries and regions.

In China, schistosomiasis is caused by *Schistosoma japonicum*, which is regarded as a major parasitic disease with a documented history of over 2100 years. In the 1950s, the population of China was approximately 600 million with an estimated 11.6 million people infected [9]. The government has taken some highly effective and comprehensive strategies to reduce the rates of transmission of schistosomiasis since the 1950s [10]–[12]. By 2011, the number of infected people had been reduced to an estimated 280,000 [13]. Despite these sustained efforts and achievements, there are still many major challenges such as patient susceptibility to infection and re-infection, the effects of global warming, increased population mobility, the existing extensive snail habitats with complicated environments, ecosystem changes caused by the construction of the Three Gorges Dams and the South–north Water Conversion Project [14], [15]. Complete elimination of schistosomiasis will take a long time and has proven to be a difficult task.

Forecasting as a form of early surveillance and detection can facilitate the development of effective control strategies for schistosomiasis. Many approaches have been adopted in forecasting infectious diseases, e.g. the exponential smoothing model [16], Grey model [17], Markov chain model [18], and ARIMA model [19]–[21]. Among these models, the linear ARIMA model is the most popular, but the accuracy of the prediction is limited by its inability to capture nonlinear relationships in the data, which is a problem because most real world applications involve nonlinear components [22]. To overcome the restriction of linear models, the artificial neural network (ANN) has been applied in many research fields, such as power and energy [23], hydrology [24], environment [25], finance and economy [26], and medicine [27]. The ANN has enhanced forecasting accuracy due to its intrinsic properties which approximate any sort of arbitrary nonlinear function [28], [29]. However, the single ANN model is unable to incorporate both the linear and nonlinear patterns found in the real world. More recently, hybrid models using ARIMA and ANN have been proposed to forecast complex events with high prediction performance [22], [30]–[33].

Therefore, we constructed a hybrid model combining ARIMA and NARNN to forecast the prevalence of schistosomiasis in humans, which provides a methodological basis for predicting and detecting schistosomiasis infection in endemic areas.

## Materials and Methods

### Data sources

The government of China has developed several surveillance programmes for controlling schistosomiasis since 1950s. These surveillance contents include the prevalence in humans, bovines, and snails; the geographic distribution of infection; meteorological and hydrological conditions; the economic status of the infected population. The surveillance results are recorded in local Center for Disease Control and Prevention (CDC). According to the National Scheme of Schistosomiasis Surveillance and the Scheme of Schistosomiasis Surveillance in Hubei Province, surveillance points have been set up by Chinese CDC and Hubei CDC complying with the following principles: surveillance points represent the schistosomiasis infection status in our country or our province; the corresponding work units are able to undertake and complete the monitoring task; endemic villages are chosen as surveillance points based on the layering principle; surveillance points remain unchanged within five years.

Qianjiang city, which is located in the south-central Hubei Province, suffers from a very high prevalence of Schistosomiasis japonica because most of the district is covered by lakes and marshes that are suitable for the breeding of snails. One national surveillance point and nine provincial surveillance points have been set up in Qianjiang City. The study chose humans as the research object without regard to bovines or snails. The monitoring method of human schistosomiasis in these surveillance points: all of residents more than 6 years old were examined and the monitoring time was from October to November at every year. The method of examination: during 1956 to 2003, residents were examined by stool Kato-Katz examination with three slides from a single stool specimen and the prevalence equaled the positive rate of stool examination; during 2004 to 2006, the government experimented the serum examination with indirect hemagglutination assay (IHA), but the positive rate of stool examination was still regarded as the prevalence; since 2007, residents were screened by IHA, then the positive individual was reconfirmed by stool Kato-Katz examination and the prevalence was equal to the positive rate of serum examination multiplied by the positive rate of stool examination. We obtained the annual report data of human prevalence of schistosomiasis from 1956 to 2012 from the Qianjiang CDC (Table S1).

### Methods

It has previously been shown that reliable prediction models combine linear and nonlinear components [31], [34]–[36]. In our study, we also adopted such method as follows:
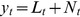
(1)


Where 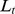, 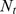 and 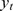 denote the linear component, the nonlinear component and the original time series respectively. In the first phase, we used the ARIMA model to generate the residual series:
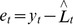
(2)


Where 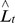 is the predicted value by the ARIMA model at time t. In the second phase, NARNN model was used to model the residual series from the ARIMA model:
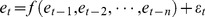
(3)


Where 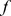 is a nonlinear function determined by the neural network and 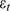 is the random error. The estimation of 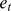 by (3) yields the forecast value, 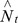.

Finally the combined forecasting values of the time series are obtained as follows:
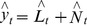
(4)


### Developing the ARIMA model to analyze the original time series

The ARIMA model consists of three main parameter [37], including the autoregressive (AR) term, the moving average (MA) term and the differencing (D) term. The AR term, 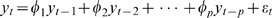, relates the observation made at year t to the previous year t-1 or earlier years. The MA term, 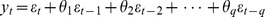, estimates the random error which is defined as the difference between the observation and forecasting value at year t to the previous year (t-1) or earlier years. The differencing is used to de-trend the time series, and D term is an integer and referred to as order of differencing. Augmented Dickey-fuller Unit Root (ADF) test is used to identify whether the time series is stationary or not. So when the series tested by ADF test is non-stationary, we can use differencing to transform it into a stationary series. The building block for time series models is that the white noise is a 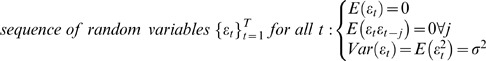


Lagged scatter-plots, autocorrelation function (ACF) and partial autocorrelation function (PACF) plots are used to identify the temporal autocorrelation in the yearly data. Model parameters are estimated by the maximum likelihood method. The parameters that have significant differences are kept and all others are excluded. The residual series needs to be inspected, which ideally shows no secular or seasonal trends as white noise [38]. The model with the minimal Bayesian information criterion (BIC) value is selected to be best fitted.

In this study, for the prediction performance comparison, the modelling data set was from 1956 to 2008 and the testing data set was from 2009 to 2012. However, we reconstructed the ARIMA model using the entire 57 year data set in order to forecast the prevalence of schistosomiasis in the future four years. This information was then used to compute the residual series by equation (2) as the target series of NARNN. We used SAS Software Version 9.2 to develop the ARIMA model.

### Constructing the NARNN model to predict the residual series

ANNs can be classified into dynamic and static categories. Static neural networks have no feedback elements and no delays, but rather they calculate output directly from the input through feed forward connections such as feed-forward, back-propagation network (FFBP), radial basis function network (RBF), or probabilistic neural network (PNN). In dynamic neural networks, the output depends not only on the current input to the network, but also on the previous inputs, outputs, or states of the network, including time-delay network (TDN), layer-recurrent network (LRN), and nonlinear autoregressive network with exogenous inputs (NARX). NARNN, which is one of the dynamic neural networks, can learn to predict a simple time series given past values of the same time series, 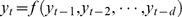. It is based on the FFBP [39] first introduced by Rumelhart, Hinton and Williams [40], which can approach any non-linear functions, and for each of these time sequences inputs, there is a tapped delay line to store previous value.

In this paper, we utilized the neural network time series tool which was one of the graphical user interfaces (GUI) in MATLAB [41]. In this tool, NARNN incorporates a default two-layer FFBP with a sigmoid transfer function in the hidden layer and a linear transfer function in the output layer with the Levenberg-Marquardtal algorithm. Using the tool, command-line scripts are generated automatically which helps simplify the construction of the model. The next section demonstrates how to train NARNN to fit the time series.

**Figure 1:**
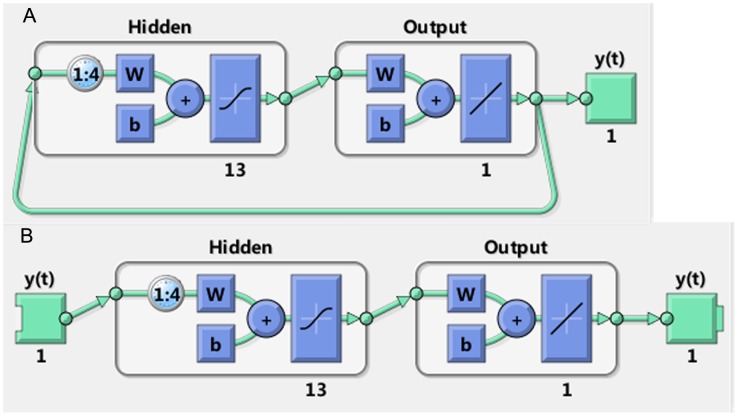
The configuration of the NARNN. The final established NARNN (original prevalence series as target series) consisted of one output layer with 1 neuron and one hidden layer with 13 neurons and 4 delays. Figure 1A shows the close loop form and Figure 1B shows the open loop form.

According to the GUI's direction, we inputted the target series and eventually obtained a command-line script, which was then modified as shown below:

- Step 1: We inputted the target series and obtain a command-line script.
- Step 2: We used the default data division function type: divide-rand, which divided the data set randomly. The data was divided three parts, the training subset used to train the network, the validation subset used to stop training before over-fitting, the testing subset used as a completely independent test of network generalization the study, the ratios for training, validation, and testing were set to 0.80, 0.10 and 0.10, respectively.
- Step 3: We adjusted the arguments feedback delays and hidden units by trial and error. The error autocorrelation plot, the time series response plot, the MSE and the correlation coefficient (R) were analyzed to choose the optimal model.
- Step 4: According to the feedback delays, we inputted the targets of the closed loop network for forecasting the prevalence of next four years. The open loop and close loop were important configurations in modelling and forecasting. In the open loop, a one-step-ahead prediction was performed. The configuration of the NARNN is showed in Figure 1. The output of the NARNN (Figure 1A), *y*(*t*), is fed back to the input of the network (through delays), since *y*(*t*) is a function of *y*(*t*-1), *y*(*t*-2), …, *y*(*t*-d). However, for efficient training this feedback loop can be opened (Figure 1B). Because the true output is available during the training of the network, it is used instead of feeding back the estimated output. This has two advantages. The first is that the input to the feedforward network is more accurate. The second is that the resulting network has a purely feedforward architecture, and therefore a more efficient algorithm can be used for training. After training, the network would be converted to closed loop form which was used for multi-step-ahead prediction.

Moreover, we constructed a single NARNN using the original prevalence series (OS) from 1956 to 2008 as the target series in order to compare with the hybrid model. The NARNN modelling was implemented using the Neural Network Toolbox in MATLAB Version 7.11(R2010b).

### Performance statistic index

In order to evaluate prediction performance, the mean square error (MSE), mean absolute error (MAE) and mean absolute percentage error (MAPE) were used to compare the forecasting capabilities of the ARIMA, NARNN and ARIMA-NARNN models:
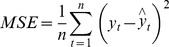
(5)

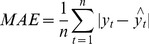
(6)

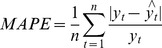
(7)


## Results

### ARIMA model

**Figure 2:**
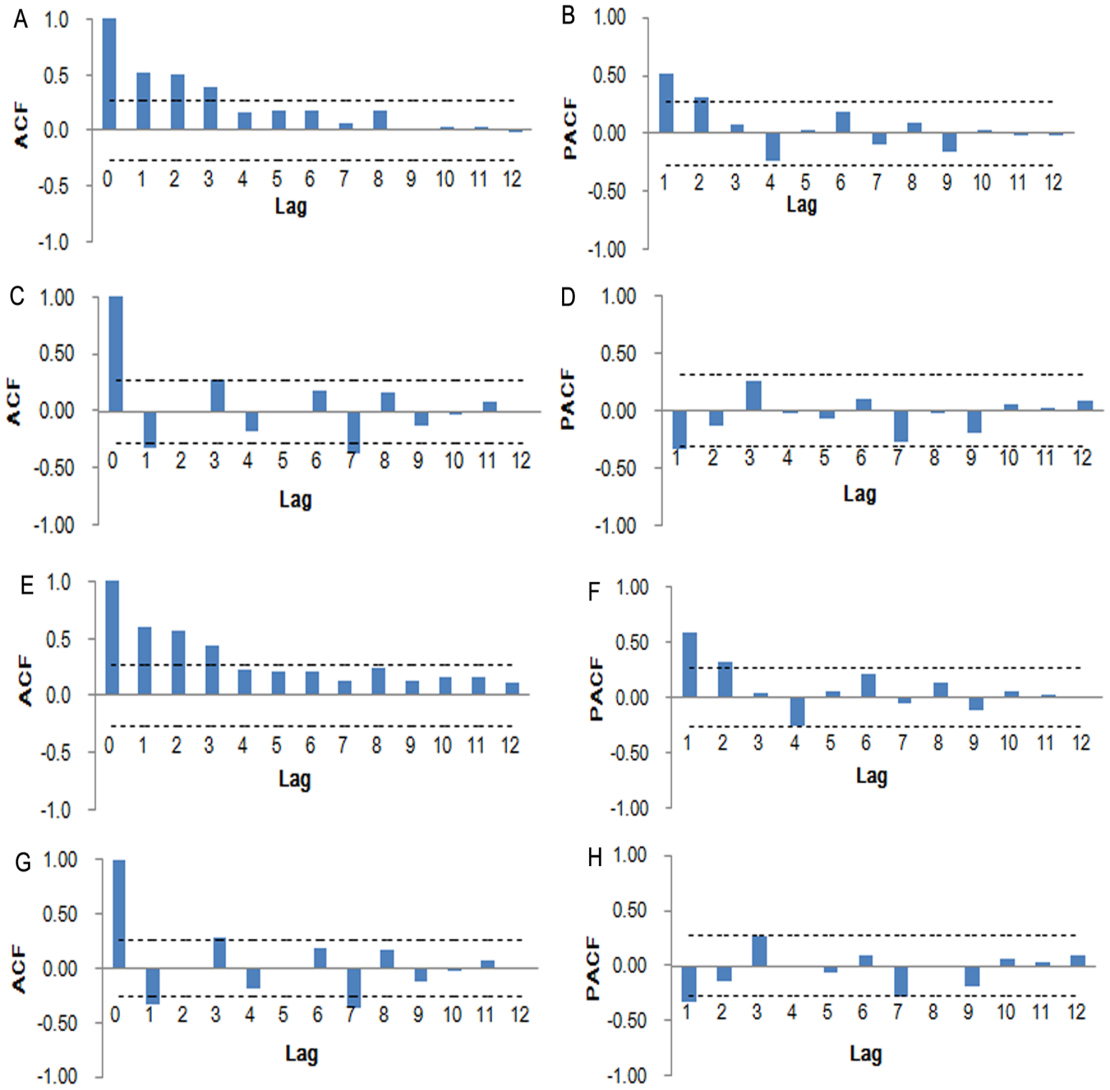
Autocorrelation function (ACF) and partial autocorrelation function (PACF) plots of original prevalence series. A and B. ACF and PACF plots of original schistosomisis prevalence (1956–2008); C and D. ACF and PACF plots after one order of regular differencing (1956–2008); E and F. ACF and PACF plots of original schistosomisis prevalence (1956–2012); G and H. ACF and PACF plots after one order of regular differencing (1956–2012). Dotted lines indicate 95% confidence intervals. Most of the correlations fall around zero within their 95% confidence intervals except for the one at zero lag, which indicate the series achieved stationary.

The original prevalence series achieved stationary after one order differencing. The ACF and PACF plots of original prevalence series are displayed in Figure 2. We found the minimum BIC (2, 0) = 0.636296. The autocorrelation check for residual is presented in Table 1. All the *P*-values>0.05 showed that the residual series was a white noise series, which indicates the information was extracted sufficiently. The results of the parameter estimation are shown in Table 2. The final mathematical model is expressed as follows:
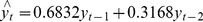
(8)

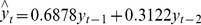
(9)


**Table 1:** The autocorrelation check for residuals in ARIMA model.

Lag	A		B	
	*Χ^2^*	*P-value*	*Χ^2^*	*P-value*
6	6.08	0.2981	6.63	0.2500
12	13.13	0.2849	14.35	0.2144
18	16.25	0.5059	17.78	0.4030
24	21.71	0.5378	23.38	0.4386

Note: A = modelling data from 1956 to 2008, B = modelling data from 1956 to 2012.

**Table 2:** The parameter estimation of ARIMA model for two modelling data set.

Modelling set	Parameter	Estimate	Standard error	*t*-value	*P*-value*	Lag
1956–2008	AR 1,1	−0.31675	0.13308	−2.38	0.0211	1
1956–2012	AR 1,1	−0.31220	0.12811	−2.44	0.0181	2

*: Parameter estimation was considered statistically significant (*P*<0.05).

(8)Prevalence data from 1956 to 2008 as the modelling data set.

(9)Prevalence data from 1956 to 2012 as the modelling data set.

**Figure 3:**
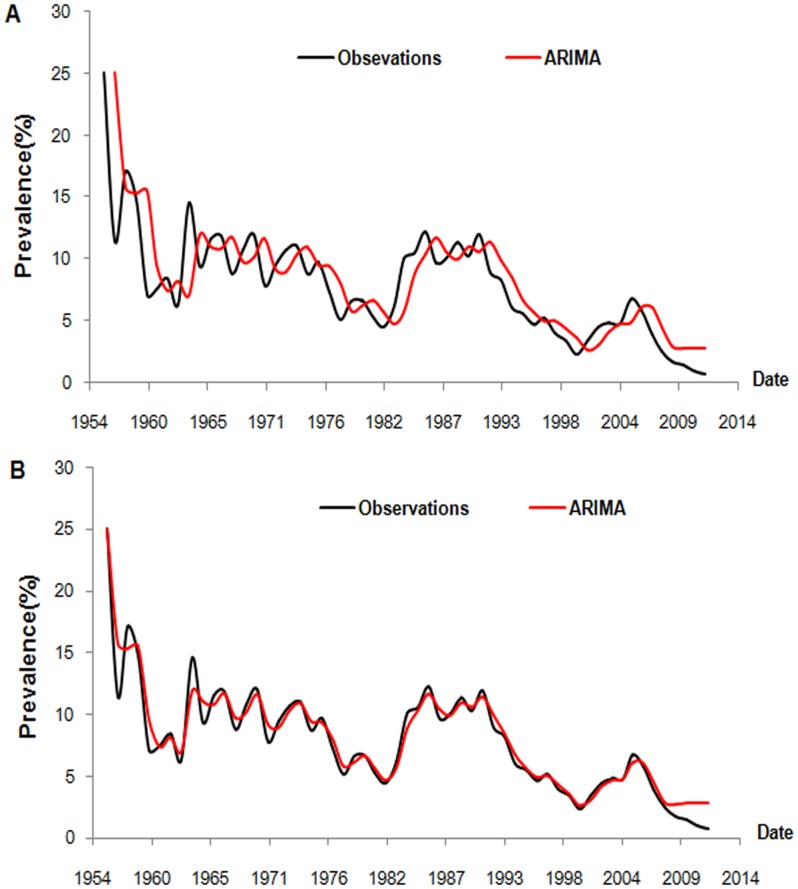
Prevalence of schistosomiasis and its predicted values from the ARIMA model. When we completed the ARIMA modelling (1956–2008) and drew the prediction curve (Figure 3A), we discovered that it made the model fitting better to advance the prediction series with one lag period (Figure 3B).

The prediction curve showed a similar trend, but with a lag, compared to the observation curve (Figure 3A) from 1956 to 2008. Therefore, we advanced the prediction series by one lag period (Figure 3B), and then computed the residual series (RS), which was subsequently used as the target series of NARNN. The same computations were performed on the prediction series from 1956 to 2012, and the result was termed the new residual series (NRS). The predicted values of the testing set, which spanned from 2009 to 2012, were 2.74%, 2.79%, 2.77%, and 2.78% respectively. The predicted prevalence in the future four years, ranging from 2013 to 2016 were 0.75%, 0.76%, 0.76%, and 0.76% respectively.

### NARNN model

**Figure 4:**
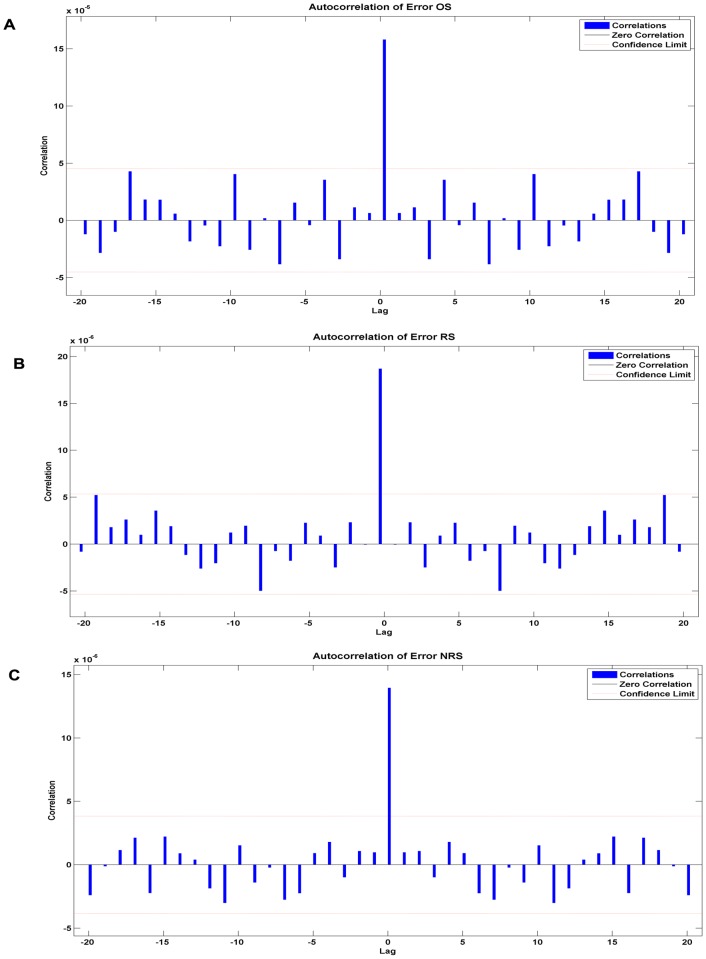
Error autocorrelation plots of different target series. The error autocorrelation was one of the evaluation parameters in the modelling process. As shown in the figure, the correlations except for the one at zero lag, all fall within the 95% confidence limits around zero, which demonstrates that the model reliably corresponds to the data.

**Figure 5:**
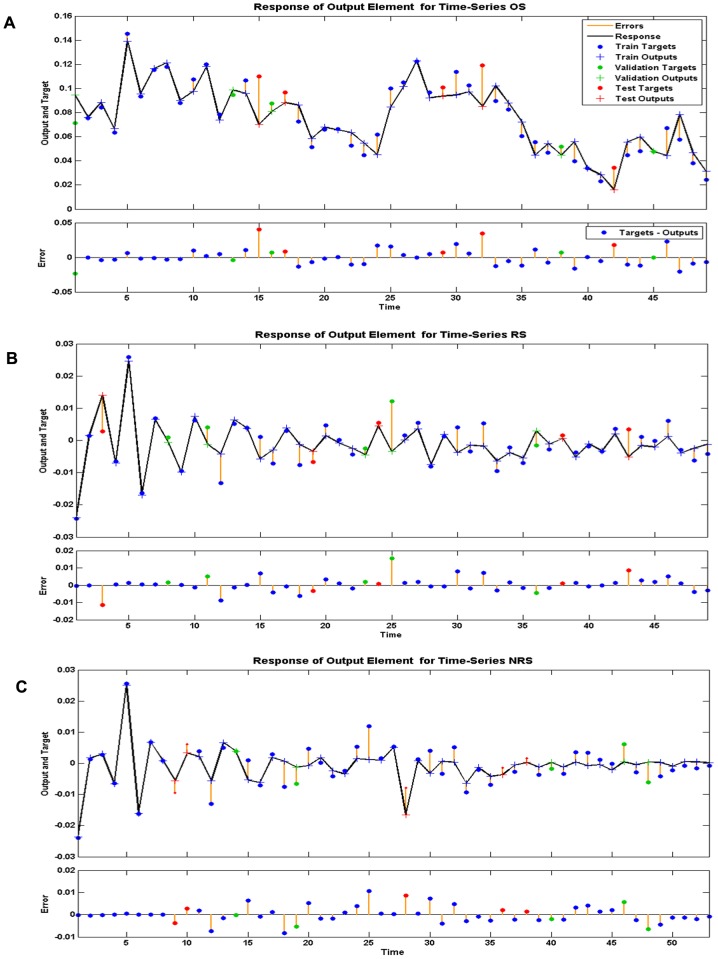
The time-series response plots of different target series. The plots indicate which time points are selected for training, testing and validation. Since the outputs were distributed evenly on both sides of the response curve and the errors versus time were small, we determined that we had chosen the appropriate model.

The most appropriate network we found applied to forecast these target series had the number of hidden units and delays, the MSE and R as shown in Table 3. All the R values were greater than 0.8. The network diagram (original prevalence series as target series) is an example to illuminate the configuration of NARNN and presented in Figure 1. The error autocorrelation function plot of different target series is displayed in Figure 4. The correlation coefficients for all the models except the trend with zero lag fell within the 95% confidence limits. The response of the network, outputs, targets and errors versus time are displayed in Figure 5, which shows that the errors were small in the training, testing and validation sets. We observed that the predicted residuals from 2009 to 2012 were −0.94%, −0.81%, −0.38%, and −0.91% respectively, and from 2013 to 2016 were −0.0029%, 0.043%, 0.0044%, and 0.010% respectively.

**Table 3:** The optimum networks configuration of different target series.

Target series	Hidden units	Delays	MSE of training	MSE of validation	MSE of testing	*R*
**OS**	13	4	0.9908	1.3174	6.4501	0.9059
**RS**	14	4	0.1047	0.5904	0.4245	0.8274
**NRS**	15	4	0.1231	0.2147	0.2051	0.8552

Note: OS = original prevalence series, RS = residual series, NRS = new residual series

All MSE values should be multiplied by 10^−4^.

### Comparing analysis

According to equation (4), we can get the predicted prevalence from the ARIMA-NARNN model (Table 4).

**Table 4:** The predicted prevalence (%) from three models.

	2009	2010	2011	2012	2013	2014	2015	2016
**Observations**	1.71	1.45	0.94	0.70				
**ARIMA**	2.74	2.79	2.77	2.78	0.75	0.76	0.76	0.76
**NARNN**	1.84	1.87	2.42	3.57				
**ARIMA-NARNN**	1.80	1.98	2.39	1.87	0.75	0.80	0.76	0.77

Note: The 2009–2012 values are predicted using modelling data 1956–2008.

The 2013–2016 values are predicted using modelling data 1956–2012.

**Figure 6:**
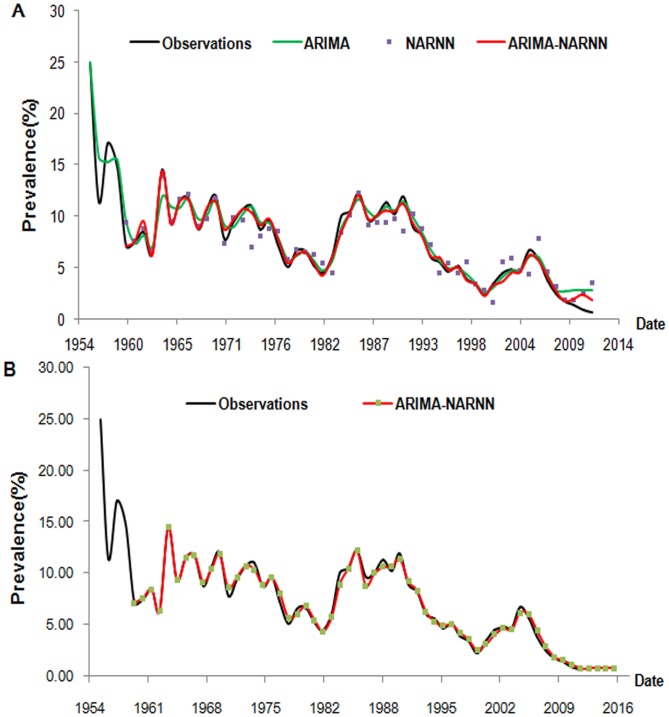
The change trend of prevalence of schistosomiasis from three models. The comparison of observation and predicted values between the hybrid ARIMA-NARNN model, and the single ARIMA or NARNN model are shown in Figure 6A. On the whole, the red line is closer to the observation curve that indicates the predicted values from the ARIMA-NARNN model are the best fit for the prevalence of schistosomiasis in humans. Figure 6B shows the predicted prevalence of schistosomiasis (1960–2016) from the reconstructed hybrid ARIMA-NARNN model.

Table 5 presents the differences in modelling error (from 1956 to 2008) and testing error (from 2009 to 2012) between the observed and predicted values using each of the three models. The hybrid model was the best with the lowest MSE, MAE and MAPE. The point-to-point comparison between observations and predicted values is given in Figure 6A. The prediction curve from the ARIMA-NARNN model was the best fit for the observed curve of the prevalence of schistosomiasis.

**Table 5:** The prediction performance results of three models.

	Modelling error			Testing error		
Model	MSE	MAE	MAPE	MSE	MAE	MAPE
**ARIMA**	0.5817	0.0056	0.0770	2.6277	0.0157	1.6095
**NARNN**	1.5812	0.0094	0.1441	2.6565	0.0123	1.5111
**ARIMA-NARNN**	0.1869	0.0029	0.0419	0.9375	0.0081	0.9064

Note: All MSE values should be multiplied by 10^−4^.

### The ultimate forecasting result

The values of forecasting from the hybrid model in the future four years, beginning in 2013 were 0.75%, 0.80%, 0.76% and 0.77% respectively. The predicted change trend (1960–2016) from the ARIMA-NARNN model is shown in Figure 6B. The modelling MSE, MAE and MAPE were 0.1395×10^−4^, 0.0028, and 0.0523 respectively. The results indicate that the hybrid model was well fitted to the data of schistosomiasis prevalence in humans.

## Discussion

To our knowledge, this study was the first to develop and apply the ARIMA-NARNN hybrid model in a parasitic disease, with the specific purpose of forecasting disease trends and guiding control strategies. We experimented with the single ARIMA model, the single NARNN model and a proposed ARIMA-NARNN hybrid model to compare forecasting performance based on the MSE, MAE, MAPE, and found that the hybrid model achieved better prediction accuracy than either of the models used separately.

The observations as shown in Figure 3 shows that the prevalence have been in a higher level (most of them more than 5%) and often fluctuate during 55 years from 1956 to 2012, but with a downward trend in the overall development. The predicted prevalence of schistosomiasis by the ARIMA-NARNN model over the next four years meet the criteria of transmission control (prevalence less than 1%) in our country [42], which indicates that our control strategies that are already in place were efficient. Nevertheless, it is still far from the transmission block criteria (no cases within five consecutive years) and elimination goal. The model predicts a no-downward trend, which underscores the importance of adjusting and enhancing control programmes to prevent rebound of the disease and further achieve the elimination of schistosomiasis. All the strategies mentioned below could be useful to adjust and enhance our control and elimination programmes: focusing on the construction of highly sensitive surveillance and response system; transferring more funds to some high endemic surveillance points to reinforce some interventions such as mollusciciding, chemotherapy of local residents and bovines, faecal management, agriculture irrigation system modification; accelerating the development of schistosomiasis diagnosis technologies; strengthening health propaganda and education.

For decades, many researchers have made efforts using mathematical models to predict patterns of schistosomiasis transmission [43]–[48] and yet the numerous influencing factors of schistosomiasis infection [1] [49]are collected uneasily that make modelling difficult. However, time series forecasting is a relatively easy and useful approach in which past prevalence is used as a model which is then used to extrapolate the time series into the future, according to its own development rules. We integrated the classical linear time series forecasting ARIMA model and the dynamic NARNN model to forecast the prevalence. In the first modelling phase, the ARIMA model was established. The mathematical formulas (8) and (9) show that the predicted value at year t depends on the previous years t-1 and t-2. Table 5 shows that the modelling errors from ARMIA model are small and fitting result is good, but the testing errors are relatively large. The modelling and testing errors from single NARNN model are also big. In the second study phase, the NARNN model was set up to forecast residuals. The number of feedback delays was set to 4 by trial and error, which indicated that the residual at year t depended on the previous 4 years values. When we combined the two models, the modelling and testing errors were decreased. The modelling MSE, MAE, MAPE decreased by 67.87%, 48.21%, 45.58% and the corresponding testing error fell by 64.32%, 48.41%, 43.68% respectively as compared to using ARIMA model alone. When compared to single NARNN model, the modelling MSE, MAE, MAPE decreased by 88.18%, 69.15%, 70.92% and the corresponding testing error reduced by 64.71%, 34.15%, 40.02% respectively. The results from this study demonstrated that our hybrid model of tracking prevalence was reasonable.

In this study, we chose NARNN to simulate the nonlinear part of the time series due to its high fault tolerance performance, dynamic property and ability to capture any nonlinear data structures without prior assumptions [26], [50]. Prior to this study, several hybrid techniques had been proposed, which used mainly the static neural networks, such as BP, RBF, PNN, or generalized regression neural network (GRNN) [30], [33], [36], [51], [52]. Dynamic neural networks, such as NARNN are generally more powerful than static networks in time series forecasting because dynamic networks have memory and can be trained to learn time-varying patterns. Furthermore, NARNN is also recommended by the MATLAB software (R2010b) since it is capable of more accurate learning than the traditional dynamic Elman network. NARNN can learn to predict a simple time series given past values of the same series which was applicable to our study data. In this paper, we used the neural network time series tool in MATLAB which allowed the calculation in an easy-to-use graphical environment to guide us to design the NARNN model. The dynamic NARNN processing was determined automatically on the best form by the software.

### Limitation of the study

There are some limitations in this study. Firstly, there are no standardized methods for determining the optimum number of hidden nodes, delays and other parameters for an ANN model. In practice they are often chosen by trial-and-error and the specific prediction process cannot be explained [53]. Secondly, determining the 95% confidence interval for the forecast is an additional problem [54]. Thirdly, the forecasting model's ability to extrapolate is limited, the longer the forecasting time, the lower the prediction accuracy. Fourthly, our study only focused on Qianjiang City. However, different groups in different living environments may have different transmission patterns and prevalence. Whether the model is appropriate for other schistosomiasis-endemic areas and other infectious diseases remains to be investigated.

## Conclusion

In summary, the ARIMA-NARNN model could be used to reliably forecast the prevalence of schistosomiasis in humans of Qianjiang City, which provides a promising methodological basis for the monitoring of schistosomiasis in the study area. Further studies are warranted to certify the feasibility of the hybrid model applied to predict schistosomiasis infection in other endemic areas or other infectious diseases.

## Supporting Information

Table S1 The original data of the study area.
(XLS)
